# The role of cultural, community and natural assets in addressing societal and structural health inequalities in the UK: future research priorities

**DOI:** 10.1186/s12939-021-01590-4

**Published:** 2021-11-24

**Authors:** L. J. Thomson, R. Gordon-Nesbitt, E. Elsden, H. J. Chatterjee

**Affiliations:** 1 Genetics, Evolution and Environment, UCL Division of Biosciences, University College London, London, UK; 2 Kings Culture, Kings College London, London, UK; 3 UCL Epidemiology and Public Health, University College London, London, UK

**Keywords:** Consultation workshop, Covid-19, Expert opinion, Health, Inequalities, Survey

## Abstract

**Background:**

Reducing health inequalities in the UK has been a policy priority for over 20 years, yet, despite efforts to create a more equal society, progress has been limited. Furthermore, some inequalities have widened and become more apparent, particularly during the Covid-19 pandemic. With growing recognition of the uneven distribution of life expectancy and of mental and physical health, the current research was commissioned to identify future research priorities to address UK societal and structural health inequalities.

**Methods:**

An expert opinion consultancy process comprising an anonymous online survey and a consultation workshop were conducted to investigate priority areas for future research into UK inequalities. The seven-question survey asked respondents (*n* = 170) to indicate their current role, identify and prioritise areas of inequality, approaches and evaluation methods, and comment on future research priorities. The workshop was held to determine areas of research priority and attended by a closed list of delegates (*n* = 30) representing a range of academic disciplines and end-users of research from policy and practice. Delegates self-selected one of four breakout groups to determine research priority areas in four categories of inequality (health, social, economic, and other) and to allocate hypothetical sums of funding (half, one, five, and ten million pounds) to chosen priorities. Responses were analysed using mixed methods.

**Results:**

Survey respondents were mainly ‘academics’ (33%), ‘voluntary/third sector professionals’ (17%), and ‘creative/cultural professionals’(16%). Survey questions identified the main areas of inequality as ‘health’ (58%), ‘social care’ (54%), and ‘living standards’ (47%). The first research priority was ‘access to creative and cultural opportunities’ (37%), second, ‘sense of place’ (23%), and third, ‘community’ (17%). Approaches seen to benefit from more research in relation to addressing inequalities were ‘health/social care’ (55%), ‘advice services’ (34%), and ‘adult education/training’ (26%). Preferred evaluation methods were ‘community/participatory’ (76%), ‘action research’ (62%), and ‘questionnaires/focus groups’ (53%). Survey respondents (25%) commented on interactions between inequalities and issues such as political and economic decisions, and climate. The key workshop finding from determining research priorities in areas of inequality was that health equity could only be achieved by tackling societal and structural inequalities, environmental conditions and housing, and having an active prevention programme.

**Conclusions:**

Research demonstrates a clear need to assess the impact of cultural and natural assets in reducing inequality. Collaborations between community groups, service providers, local authorities, health commissioners, GPs, and researchers using longitudinal methods are needed within a multi-disciplinary approach to address societal and structural health inequalities.

## Background

Reducing health inequalities in the UK has been a policy priority for over 20 years [1], yet, despite efforts to create a more equal society, progress has been limited [2]. Some areas of inequality have widened [3], particularly during the Covid-19 pandemic [4]. Considerable research on the existence and prevalence of UK societal and structural inequalities, and their effects on mental and physical health outcomes has been reported. A survey of over 2000 working-age adults found that 75% within the lowest household income bracket experienced a mental health issue compared with 60% in the highest [5].Socio-economic disadvantage and the chronic distress it causes for adults and children has negative effects on the body’s physiology [6–8]. Analysis of data from the English Longitudinal Study of Ageing showed that older people living in deprived neighbourhoods were significantly more likely to experience mobility difficulties than those in less-deprived neighbourhoods [9].

Research has recognised that health is a product of the interdependence between humans and environmental determinants ranging from ‘provision of the ecosystem services of food, water, and air, to more nuanced stress-reducing and social capital services, to the role of forests in mitigating the health threats posed by climate change’ ([10]:1006). From an ecological viewpoint, researchers theorised that public health should address four dimensions consisting of ‘material’ referring to physical building blocks on which life depends; ‘biological’ involving bio-physiological processes including animal and plant species; ‘cultural’ concerning interpersonal relationships, community and family traditions; and ‘social’ related to institutions between people in terms of laws, social arrangements, conventions, and frameworks ([11]:3). To explore underlying mechanisms linking urban environments to public health and social equity, four principles for an ecological public health model were proposed comprising ‘conviviality’, ‘equity’, ‘global responsibility’ and ‘sustainability’ ([12]:528).

The Commission on the Social Determinants of Health conjectured that health inequalities were caused by ‘unequal distribution of power, income, goods, and services, globally and nationally’ which resulted in ‘unfairness in the immediate, visible circumstances of people’s lives’ ([13]:1). The chair of the Commission, Professor Sir Michael Marmot, examined the relationship between socioeconomic position and health in England and determined that two of the six principles for tackling health inequalities should be to ‘create and develop healthy and sustainable places and communities’ and to ‘strengthen the role and impact of ill health prevention’ with a focus on an asset-based approach ([14]:1). A key report advocated a move towards transdisciplinary ‘health of the public research’ involving disciplines ‘that would not usually be considered to be within the public health field; an approach integrating aspects of natural, social and health sciences, alongside the arts and humanities, which directly or indirectly influence the health of the public’ ([15]:5).

As indicated above, it is feasible that cultural assets involving arts and humanities, social engagement and sense of place within communities, and environmental determinants such as natural assets could be used to improve public health and tackle societal and structural health inequalities. As these inequalities have widened in the UK due to Covid-19, there is a current gap in the knowledge base regarding which areas of inequality to prioritise, particularly where value for money is important given limited financial resources. The aim of the current study was to use an expert opinion consultancy process to determine the most pressing inequalities in the UK, and to consider future research priorities. Potential areas of inequality for the survey questions and workshop discussions within the consultancy process were based upon existing research considering the efficacy of non-medical interventions to health and wellbeing, particularly in terms of engagement with culture, community and natural assets.

Data gathered from over 15,000 UK respondents found that cultural engagement made the highest contribution to wellbeing in later life followed closely by physical activities and thinking skills [16]. A review of 900 publications linking evidence from the arts to improved health and wellbeing identified two themes: ‘prevention and promotion’, in which the arts could ‘affect the social determinants of health’ and ‘encourage health-promoting behaviours’; and ‘management and treatment’, in which the arts could ‘help people experiencing mental illness’ and ‘help to support people with neurodevelopmental and neurological disorders’ ([17]:7–8). A study conducted within deprived London communities found that, out of those engaged with the arts, 82% enjoyed greater wellbeing, 79% ate more healthily, and 77% engaged in more physical activity [18]. Arts engagement can also be effective in compensating for work-related stress [19]; a USA study of activity outside of work determined that organisations might ‘benefit from encouraging employees to consider creative activities in their efforts to recover from work’ ([20]:1). A study exploring cultural interventions beyond the UK determined that ‘artists need to critically engage with the big issues of the day – ageing populations, social isolation, addictive behaviours, substance abuse, obesity and mental ill health – all of which are underpinned by inequality’, while questioning whether access to the arts could be increased ‘without resorting to models that perpetuate inequalities’ ([21]:186).

Depression and isolation follow the social gradient, where those of lower socioeconomic status are more severely affected than those from higher socio-economic groups [14] and, in addition to affecting adults, can impact negatively upon the lives of children [14]. Several researchers have shown, however, that childhood engagement with the arts and literature can foster early physical, cognitive, linguistic, social and emotional development [22–25]. Arts engagement ‘helps to mitigate the effects of an adverse environment… enabling self-expression and empowerment and overcoming social isolation’ ([26]:10). A ten-week study of mothers singing with their babies showed faster recovery from postnatal depression, greater decrease in stress hormones, and more improvement in mother-infant bonding compared with controls using other forms of social interaction [27]. An independent study for the Welsh Government acknowledged that arts engagement aided literacy and numeracy and helped to bridge the attainment gap, but that access to these benefits was unevenly distributed [28]. An Australian study found that ‘arts education not only has intrinsic value, but when implemented with a structured, innovative and long-term approach, it can also provide essential extrinsic benefits, such as improved school attendance, academic achievement across the curriculum as well as social and emotional wellbeing’ ([29]:3).

Unequal access to resources as a source of social stratification has long been recognised by sociologists [30], and the possibility that social inequalities are magnified and reinforced through differences in communities has become an important theme [31]. A qualitative review of place and space across the life course highlighted that ‘development and perception of community has a role to play in individual and group wellbeing’ ([32]:24). It was further recognised that the poorer health of economically deprived communities could be explained by low social status but ‘offset by a sense of community, by a sense of identity’ ([33]:78). The boosting of social relations was described as a ‘key ingredient of both individual and community wellbeing’ ([34]:5). The finding aligned with an asset-based rather than deficit-based model in which assets are regarded more broadly than at the level of an individual by local government [35]. The importance of maintaining meaningful participation in later life ‘through social, creative or physical activity, work, or belonging to some form of community group’ was found to contribute more than 20% of wellbeing ([36]:12). Consequently, improving a sense of community, defined as the ‘measure of a person’s integration and meaningful communication with their community, family and friends’ was seen to help to ameliorate social isolation [37].

With a view to addressing inequalities and improving communities, the Marmot Review acknowledged the importance of the green infrastructure, proposing that ‘Access to good quality air, water, food, sporting, recreational and cultural facilities and green space all contribute to reducing inequalities as well as helping to create sustainable communities’ ([14]:26). Areas of research examining the relationship between nature and health have included air quality, social cohesion, stress reduction and physical activity [38]. A study of more than 345,000 people found that, after controlling for socioeconomic status, the prevalence of 11 disease categories was at least 20% greater for those living in less green residential spaces [39]. Green spaces have the potential to address long-term health conditions linked to chronic stress and lifestyle [40], with ‘greenness’ seen as protective against adverse mental health outcomes, cardiovascular disease, and mortality’ ([41]:131). Even short physical engagements with nature appear to boost mood and self-esteem, additionally enhanced by the presence of water [42]. A review of seven UK studies [42–49] observed a statistical association between greater access to green space and improvements in mental health outcomes [50]. Regular weekly use of a natural environment was associated with a 43% lower risk of poor general health [46]. People who moved from less green to greener areas had significantly better mental health scores in the three years following the move than previously [43]. People with a high amount of local green space appeared less affected by stressful life events than those with a low amount within the same 3Km radius [51]. Gardening was found to promote relief from acute stress, as assessed by salivary cortisol [52]. Although a number of explanations have been offered for the association of nature with health improvements, it seems plausible that being in green and natural environments enhances immune functioning [53].

As trees and other vegetation mitigate air pollution generated by road traffic and industry through carbon capture [54], it is interesting to note that the ‘most affluent 20 per cent of wards have five times the amount of parks or general green space (excluding gardens) per person than the most deprived ten per cent of wards’ ([55]:7). Conversely, the most deprived urban communities tend to experience the poorest air quality [56], with increasing risk of cancer, asthma, heart disease, dementias, mortality, and hospital admissions [57]. In addition to air pollution from traffic, noise pollution can also threaten human health [58], although well-designed urban green spaces can buffer noise and negative perceptions of it [59]. Despite the perceived benefits of green space, people from deprived urban backgrounds appear to engage less with nature than those in more affluent areas [60]. Furthermore, people from higher socioeconomic groups tend to be more physically active in their leisure time than lower socioeconomic groups [61]. However, research shows a disproportionately positive association of engagement with natural resources and wellbeing for communities at the lower end of the socioeconomic gradient [62]. A study of over 165,000 adults across England found a relationship between access to green space and walking in all socio-economic areas, whereas the relationship between green space and reduced mortality was only apparent in the most deprived areas [63]. Income deprivation in England has a weaker association with all-cause and circulatory disease mortality among people living in areas with relatively large amounts of green space than for those in less green areas [47]. Additionally, researchers found that ‘inner urban areas, which tend to have a lower quantity of green space, also tend to have a higher proportion of black and minority ethnic communities’, and recognised that ‘the results are intimately related to the circularity of disadvantage – black and minority ethnic communities are more likely to be living in areas of deprivation which have markedly less green space than average’ ([64]:14).

Inequalities across the UK have been amplified by the impacts of Covid-19 on health and wellbeing and have ‘not been felt uniformly across society’ ([65]:7). Furthermore, ‘many already deprived communities have faced even greater hardship and loss of assets and resources’ ([4]:1). Covid-19 has exacerbated existing structural and social inequalities, with ‘particularly negative health outcomes for those already disadvantaged in society’ ([65]:7). The pandemic has heightened awareness of chronic conditions associated with poverty and the greater likelihood of mortality; ‘fallout from the pandemic threatens to expose – and widen – inequality in brutal fashion’ ([66]:4). Analysis of Covid-19 data (Apr–Jul 2020) showed that ‘age-standardised mortality rate of deaths involving Covid-19 was 3.1 deaths per 100,000 population for the most deprived areas in England in July; statistically significantly higher than the 1.4 deaths per 100,000 population in the least deprived areas’ ([67]:16). Risk of dying among those diagnosed with Covid-19 was also ‘higher in males than females; higher in those living in the more deprived areas… and higher in those in Black, Asian and Minority Ethnic groups…’ ([68]:4).

In the nationwide effort to reduce contact and to control the spread of Covid-19, the number of people experiencing loneliness as the ‘state of being without any company or in isolation from the community or society’ has inevitably increased ([69]:526). Even in the absence of a pandemic, documented evidence shows that that long periods of isolation have a detrimental effect on mental wellbeing [70]. Additionally, loneliness can be an independent risk factor for sensory loss, connective tissue and autoimmune disorders, cardio-vascular disorders, and obesity [69]. A UK survey in early lockdown (April 2020) found that 24% of adults experienced feelings of loneliness compared with 10% before lockdown, with 44% of younger adults (aged 18–24) feeling lonely during lockdown, compared with 16% before lockdown [71]. Nationally representative survey data from more than 15,000 UK respondents documented a high prevalence of general psychiatric disorders (29.20%) and loneliness (35.86%) during the pandemic, and found that people with current or past Covid-related symptoms or disadvantaged socioeconomic backgrounds were at higher risk of general psychiatric disorders and loneliness [72].

There is growing pressure for research to tackle the wider social determinants of health across developed countries through the implementation of appropriate interventions [73], but the problem is that there is an apparent lack of consensus among researchers as to which interventions are most likely to address health inequalities [74]. A report outlining nine proposals from research experts, each recommending one intervention to reduce health inequalities at a local level, targeted the living wage; life chances in childhood; lower speed limits; health-related unemployment; participatory budgeting; further and adult education; health inequalities and ethnicity; conditions for public sector workers; age-friendly urban environments; and cost-effectiveness [75]. The authors, however, did not consider consensus among the broader research community for these proposals. Other authors used a two-stage survey, involving the extent which respondents believed proposals taken from multiple sources would be effective and assessing shortlisted proposals, to determine policies to reduce UK health inequalities [76]. Recommendation through expert opinion showed some consensus, including: taxation supporting those lower down the social gradient and reducing wealth inequalities; a minimum income for healthy living; greater investment for vulnerable populations, and tackling long-term unemployment. There were, however, differences between expert opinion and recommendations based on available evidence for interventions such as smoking cessation, alcohol pricing and speed limits.

Potential interventions have been categorised into four areas, from ‘strengthening individuals, to strengthening communities, to improving living and working conditions and associated access to essential services, to promoting healthy macro-policies’ ([77]:474). It is clear that ‘turning these demands for better evidence about interventions around the social determinants of health into action requires identifying what we already know and highlighting areas for further development’ ([73]:284). The current study used expert opinion to investigate priority areas for future research into UK inequalities and to ascertain suitable methods for addressing these inequalities.

## Methods

### Design

The design used mixed methods to analyse quantitative and qualitative data from an online survey and a consultation workshop.

### Participants

Participants (*n* = 200) comprised a convenience sample of adult survey respondents (*n* = 170) and a purposive sample of consultation workshop delegates (*n* = 30). The survey was targeted at academics and researchers; voluntary, third sector, health and social care, and creative and cultural professionals; parliamentarians, policy makers, and local authority employees, who were contacted through mailing lists of research partners, comprising universities, health and social care, arts, heritage, nature, and third-sector community organisations.. Workshop delegates represented a range of academic disciplines and end-users of research from policy and practice.

### Materials

The anonymous seven-question survey ‘Inequalities in the UK: Future Research Priorities’ comprised six structured questions, with additional free-text boxes for comments, followed by an open, unstructured question (Table 1). For the ‘Inequalities in the UK’ workshop, flipchart pages were pre-printed with the categories for the activities and participants made their contributions anonymously. An online or printed privacy statement was made available to all participants for the survey and workshop.

**Table 1:** ‘Inequalities in the UK: Future Research Priorities’ survey questions

1. Which of the below best describes your current role? *[Options: Academic; Clinical Commissioner; Creative and cultural professional; Economist; Funder; Healthcare professional; Local authority employee; Parliamentarian; Policymaker; Researcher; Social care professional; Voluntary / third sector professional; and Other]*	
2. Please identify which of these key areas of inequality would benefit from more research (select all that apply). *[Options: Age; Economic factors; Education; Employment; Environment; Ethnicity; Gender; Health; Living standards; Regional; Social care; and Other]*	
3. Please rank the below areas in order of your top 3 research priorities (please indicate other areas you consider are also important). *[Options: Access to creative and cultural activities; Access to nature / outdoor spaces; Alcohol / drug / substance abuse; Child abuse; Childcare; Community; Crime; Education / training; Foodbanks; Gambling; Gentrification; Healthcare; Homelessness; Loneliness and isolation; Policing; Pollution; Public transport; Sense of place; Smoking cessation; Social care; Urban regeneration]*	
4. Please rank which of the approaches below would benefit from more research in relation to addressing inequalities (please indicate other areas you consider are also important). *[Options: Advice services; Adult education / training; Art galleries / museums / archives / historic / heritage sites; Arts (incl. performing and digital arts); Clubs / societies; Community spaces: Creative arts therapies; Early years’ provision; Green / blue outdoor spaces; Health / social care including mental health; Horticulture; Libraries; Social prescribing; Third sector / charities; Urban regeneration; Volunteering]*	
5. Please identify which geographic areas below are a priority for research (select all that apply). *[Options: East of England; East Midlands; London; North East; North West; Northern Ireland; Scotland; South East; South West; Wales, West Midlands; Yorkshire & the Humber]*	
6. Please indicate which research or evaluation methods are most appropriate for conducting research into inequalities (select all that apply). *[Options: Action research; Arts-based methods; Brain scanning techniques; Community / participatory methods; Ethnographic studies; Longitudinal studies; Meta-analysis; Physiological / biological research; Randomised controlled trials; Translational research; Quasi-experimental research; Questionnaires / focus groups and Other]*	
7. Please use this space to make any other comments about future research priorities in relation to addressing inequalities.	

### Procedure

The survey was conducted over 10 weeks (Jan–Mar 2020). Prior to beginning the survey, respondents were required to confirm that they had read the privacy statement. Following the first question about their current role, the five questions that followed asked respondents to identify and rank key areas of inequality, approaches and evaluation methods. The seventh question asked respondents to suggest other research priorities to address inequalities. Respondents took an average of 15 min to complete the survey. The consultation workshop was held at University College London over one day (Feb 2020) and attended by a closed list of delegates who gave consent to participate in the research on acceptance of the invitation. In the workshop, delegates self-selected one of four breakout groups on the understanding that they did not sit with anyone they knew, to take part in two activities. For Activity 1, ideas were brainstormed around research priority areas with all groups working on four categories of inequality: ‘health’, ‘social’, ‘economic’, and ‘other’, reflecting commonly occurring themes determined by analysis of free-text comments from survey questions two to seven. For Activity 2, each group was given a hypothetical sum of funding (half, one, five or ten million pounds) and asked how they would allocate it to their chosen priorities. For both activities, ideas were captured by group members appointed as scribes who noted responses on the flipchart pages and fed back outcomes for wider discussion.

### Analysis

Responses were analysed using mixed, quantitative and qualitative methods. Quantitative survey data were analysed using descriptive statistics, and data from key survey questions (2–4) were analysed in IBM SPSS v.25 using inferential statistics. Qualitative data comprising survey free-text comments and consultation workshop responses were analysed using deductive thematic analysis.

## Results

### Survey findings

Question 1: A third of respondents described their current role as ‘academics’ (33%) and a third as ‘voluntary/third sector professionals’ (17%) and ‘creative/cultural professionals’ (16%). The remaining third consisted of healthcare professionals’ (8%), with other roles (policymaker, researcher, local authority employee, funder, social care professional, and parliamentarian) less than 5% each.

**Fig. 1:**
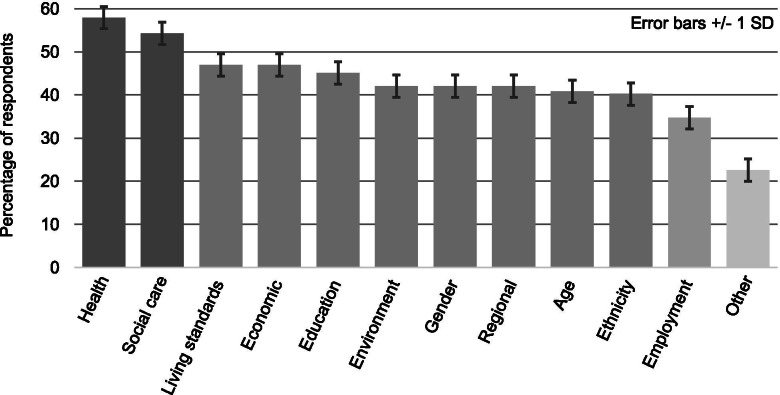
Key areas of inequality that would benefit from more research

Question 2: Respondents identified the highest key area of inequality that would benefit from more research as ‘health’ (57.93%), followed by ‘social care’ (54.27%), ‘living standards’ and ‘economic factors’ (both 46.95%), and ‘education’ (45.12%) (Fig. 1). A Chi Square goodness-of-fit test using IBM SPSS v.25 found that the observed frequency of responses was highly significantly different from that expected by chance alone, Chi^2^(11) = 33.71, *p* < .001 (two-tailed). Respondents’ comments covered three main themes: i) the integrated nature of these areas: “Inequalities are multifaceted and interconnected and cannot be divided into neat segments” and “None of these things is in isolation, each one has a knock-on effect on the others”; ii) health inequality as compounded by racial inequality: “Black, Asian and Minority Ethnic health, especially mental health is a top priority and the effects of poverty and education are determinants of these inequalities”; and iii) unequal access to public services: “In terms of inequalities, ten years of austerity have brought havoc to basic services and inequalities of access and provision have deepened and we do not know enough about impacts”.

**Fig. 2:**
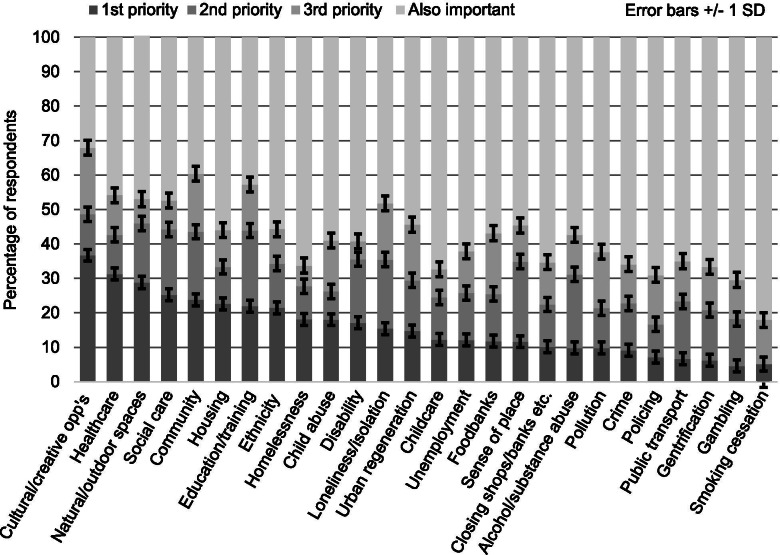
Top three research priorities and areas that are also important

Question 3: Respondents were asked to rank priorities for research and indicate which areas they thought were also important, though not a current research priority. For each area, respondents had the option of selecting one of four boxes or making no selection; boxes were labelled ‘first priority’, ‘second priority’, ‘third priority’ and ‘also important’. For research, the first priority was ‘access to creative and cultural opportunities’ (36.70%) followed by ‘healthcare’ (31.25%) and access to nature/outdoor spaces’ (28.80%); second priority was ‘sense of place’ (23.26%) followed by ‘education/training’ (21.95%) and ‘alcohol/drug/substance abuse’ (21.31%); and third priority was ‘community’ (16.83%), followed by ‘pollution’ (16.39%) and ‘loneliness and isolation’ (16.36%) (Fig. 2). Areas considered ‘also important’ were ‘smoking cessation’ (82.05%) followed by ‘policing’ (69.05%), ‘gentrification’ (66.67%) and ‘homelessness’ (66.27%). Although these four areas received a higher frequency of response than the research priorities, this finding was expected as respondents’ selections were split over fist, second and third priorities, consequently ‘also important’ areas received approximately three times as many selections as each priority area. A Chi Square test of association found that if all areas of potential inequality were included, the observed frequency of responses was not significant, Chi^2^(50) = 78.16, *p* < .007 (two-tailed), whereas if the 50% of responses with the highest frequency were analysed, findings were significant, Chi^2^(24) = 38.85, *p* < .028 (two-tailed) suggesting that responses significantly different from chance were only evident for areas with higher frequencies of response. Respondents saw that culture has “a crucial role to play in contributing a sense of place, belonging and social connections for local people”, pointing out that opportunities to engage in cultural activities were limited by sociodemographic and environmental factors: “Whilst there is much work on green spaces and cultural venues like museums, there is less on taking an holistic approach to the local neighbourhood and environment… where the air and outdoor quality of space and the character of neighbourhoods (dereliction of buildings, lack of services and cultural opportunities) all affect perceptions and vibrancy and promote inequality”.

Question 4: Similarly to Question 3, respondents had the option of selecting one of four boxes for ‘first’, ‘second’ or ‘third’ priorities and ‘also important’. Respondents ranked approaches that would benefit from more research in relation to addressing inequalities; first priority was ‘health/social care including mental health’ (54.70%), second priority was ‘advice services’ (33.96), and third priority was ‘adult education/training’ (25.61%). Also important were ‘horticulture’ (63.04%), ‘clubs/societies’ (59.57%), and ‘volunteering’ (59.32%). A Chi Square test of association found that the observed frequency of responses was highly significantly different from that expected by chance alone, Chi^2^(32) = 87.25, *p* < .001 (two-tailed). Respondent comments endorsed the first choice: “Healthcare is a top priority, coupled with an ageing population this impacts social care and this cannot be achieved without the third sector being involved in the design and delivery of social prescribing”. Comments implied that structural and economic changes were needed to alleviate negative impacts on health and social inequality: “Why are there not more options focused at a structural level? For instance, we need more research on how communities can influence local and national policies. We need more research on how consumerism drives inequalities”.

Question 5: Respondents identified the North East (61.01%) as highest priority for research followed by the North West (46.54%), Wales (41.51%), and Yorkshire and the Humber (36.36%). The South East was lowest priority for research (17.61%), followed by London (22.64%). Comments showed that other priorities were “Coastal places and regions… as these areas are often both deprived and threatened by climate change”; and specific suburban areas for example: “Scotland, in particular the areas outside of Glasgow (e.g. Dundee); Northern Ireland, in particular areas outside of Belfast”.

**Fig. 3:**
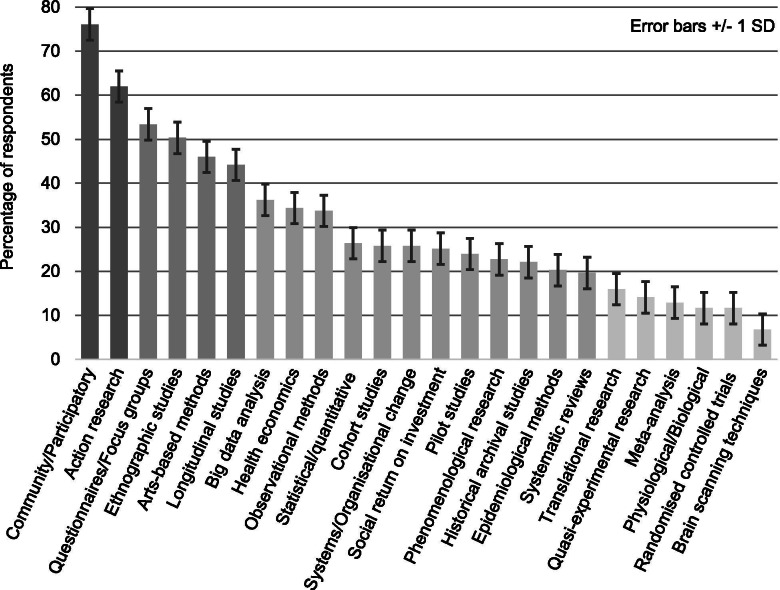
Research/evaluation methods appropriate for conducting research into inequalities

Question 6: Respondents selected ‘community/participatory’ methods (76.07%) as most appropriate for conducting research into inequalities followed by ‘action research’ (61.96%), ‘questionnaires/focus groups’ (53.37%) and ‘ethnographic studies’ (50.31%) (Fig. 3). Methods deemed less appropriate were ‘meta-analysis’ (12.88%), ‘physiological/biological research’ (11.66%), ‘randomised controlled trials’ (11.66%) and ‘brain scanning techniques’ (6.75%). Comments emphasised mixed methods: “big broad data fleshed out with narrower qualitative methods”, stressing individual experience and translational research: “methodologies that delve into people’s experience to better understand the mechanisms of change. We also need to think about narrative research that enables us to understand complexity and also translate it to wider audiences”.

Question 7: A quarter of respondents (25.40%) made comments on future research priorities that concerned interactions between inequalities and key issues: “Linking inequalities to political and economic decisions” and “inequalities that might be exacerbated by climate”. It was pointed out that health, particularly mental health, interacted with economic inequality: “stark inequality around the fact that any form of psychotherapy is very hard to access unless you can afford to pay privately”. Respondents focused on diversified research that observed individual experiences and population norms: “Interdisciplinary multi-method approaches are needed with wide geographical remits” and “explore the complexity of multiple inequalities – because inequality is multifactorial and aggregative.” Co-participatory and co-production research was highlighted as important alongside citizen researchers and making research methods more transparent: “the participant experience and whether the process can be beneficial to the communities in which it takes place” and “effective approaches draw on co-production, working directly with communities most affected by these issues. Action research… is a key priority”. These responses, plus those made in additional comments to Questions 2–5 were analysed to explore structural and systemic inequality under themes of health, social, economic, environmental, and intersecting inequalities (Table 2).

**Table 2:** Thematic analysis of structural and systemic inequality from survey comments

Theme	Codes	Examples of quotes
Health inequalities	Barriers Employment Mental health Physical health Access to services	“Access to services causes a huge disparity e.g. low-level mental health service often closed to those with co-morbidities; work status often causes disparity e.g. adults of working age have difficulties getting healthcare appointments or being involved in research as usually scheduled during the day.”
Social inequalities	Community Education Gentrification Isolation/loneliness Race and ethnicity Access to services Access to social prescribing	“…inequalities in relation to cultural provision and education, lack of cultural democracy.” “The reduction in community services reduces local support, isolates people.” “I have included gentrification and loneliness and isolation, as they are key aspects that affect community.” “A key issue for social prescribing research is whether it increases social inequalities. Wealthier areas have more community organisations that patients can be referred to.”
Economic inequalities	Community Childhood/parenting Employment Infrastructure Isolation/loneliness Economic opportunities Access to services Volunteering	“Social and economic inequalities, lack of investment in early years to support young families on low incomes. Closing libraries, post offices, reducing public services (including police, health care and transport), isolating people and dismantling communities.” “I think volunteering offers a potential route into employment for those who face barriers to employment and also has potential to reduce inequalities by bringing together different parts of the community.”
Environmental inequalities	Access to nature Eco-literacy Green space Infrastructure Living standards	“I can’t prioritise these issues. Suffice to say that I think the whole of our culture needs serious re-examination. I believe that we need to reconnect with nature, stop prioritising money as a solution to everything; develop a kinder and more compassionate society. I also believe that we need to decentralise power as much as possible.”
Intersecting inequalities	Barriers Community Employment Industry Infrastructure Living standards Mental health Physical health Transport Access to services	“All are important, and also they intersect.” “… it is so hard to separate out specific disparities as each has an impact on others and can being excluded for one thing can lead to exclusion from others. For example, working a low-paid job may lead to financial issues, which may mean no access to personal transport and so being reliant on public transport, which in turn may cost more and be more disruptive of normal life, impacting on physical and mental health.” “We need more research that looks at the social determinants of health and how these are driven by policy and industry”

### Workshop findings

Activity 1 Delegates chose not to separate research priority areas but instead discussed how inequality in one area could affect another. The key finding was that health equalities could only be achieved by tackling societal and structural inequalities, chiefly environmental conditions and housing, and having an active prevention programme (Table 3). Delegates felt that improved access to services, culture, and engagement would lead to a fairer society with shared public benefits, and that mixed methods research was vital for addressing these.

**Table 3:** Thematic analysis of responses from the consultation workshop

Research priority areas	Response themes
Social	Access to services, especially youth services Asset mapping Fundamental shift in ideology and approach of society to address structural inequalities Homelessness Instability of community and reducing social networks Isolation/loneliness Migrants Offenders Older population Orchestration of social connectivity opportunities Psychosocial crisis points: precarious work and working poor Sustainable social prescribing Social infrastructure
Economic	Accessibility: transport, disability Affordability: enabling individuals to participate. Challenging funding culture Economy of wellbeing Facilities, amenities and digital tools Fairness and shared public benefit through improved access to services Participatory budgeting Regeneration via arts spaces: theatres, dance, art, choirs
Health	Activity/physically healthy lifestyle Address structural inequalities that cause health inequalities Complex needs Co-morbidities Dementia Holistic community care Integrative healthcare Mental health Preventative medicine Social prescribing Substance misuse
Other	Cultural value and engagement Education (neurodiversity and reducing exclusion) Environment (green space/safety) Housing/homelessness Inequality in one category effects another Research methods: longitudinal research, longer-term follow-ups and mixed methods as routine policy with subsequent funding

Activity 2 The half a million pound group aimed to invite young people from areas of known health inequality to take part in prevention programmes over two years. Participants would be supported by local voluntary agencies, general practices, and libraries, and trained in mixed methods skills to co-research sense of community, social isolation and wellbeing using creative approaches such as video documentation. The one million pound group planned to understand the community ecosystem and create a toolkit to describe inequality in relation to this by consulting those affected by inequalities. They wanted to chart community demographics in terms of health and social care and then co-design interventions to map community assets and how they should be used. The five million pound group decided to identify local authorities with non-widening or reducing inequality gaps to discover what they were doing differently. In a five-year study they planned to compare locations using a mixed-methods participatory approach to identify transferable strategies as to how conditions might enable reduction in the health gap. The ten million pound group wanted to optimise analysis of inequalities by establishing data linkage across primary care networks concerning social determinants of health. They intended to engage communities in understanding assets through joint strategic needs assessment across deprived areas, specifically identifying health, transport and place-making issues. They would offer funding to five communities and empower them to improve health outcomes. Decisions would be based on an expert facilitation process to enhance social connectedness through subsidised social activities and to provide spaces for intergenerational and interethnic community assets, such as multicultural centres

## Discussion

The expert opinion consultancy process employed in the current study identified a clear need for research to assess the impact of community interventions on reducing inequality. Participants advocated access to creative and cultural, and nature and outdoor activities, plus adult education and volunteering to strengthen individuals, with communities improved through a sense of place, urban regeneration and tackling issues such as homelessness and substance abuse. The survey showed that access to health and social care was of overriding importance in terms of essential services, and the consultation workshop findings indicated that other services, such as access to public transport, were important as lack of infrastructure could precipitate other conditions such as isolation. These findings align with identified priority areas for intervention with aims ranging from ‘strengthening individuals, to strengthening communities, to improving living and working conditions and associated access to essential services , and finally to promoting healthy macro-policies’ ([77]:474).

The current study showed consensus in that all four workshop groups determined that health, social and economic inequalities were interlinked, and the survey found that health and social care were key priorities. Similar to previous findings [1], there was less consensus in other parts of the survey, with most areas seen as important. Two of the nine proposals for tackling inequalities [75] were addressed by participants in the current study in terms of health inequalities and ethnicity, and adult education and training. Finding were in keeping with previous recommendations by expert opinion [76], particularly for the workshop discussion, in that investment for vulnerable populations, long-term unemployment, primary care, and home-building was debated in the groups. Workshop recommendations advocated a multi-disciplinary ecosystem approach akin to an ecological public health model integrating biological, social, and cultural aspects of public health [11]. Studies might involve the co-production of new interventions with local residents, or draw on the work undertaken by local authorities in providing individualised solutions through site visits and discussions with residents, wardens, management companies and developers, such as Wigan’s five-year plan to get residents in shape, or Halton’s Citizens’ Advice Bureau providing fast-track support to parents with young children [35].

The aims of the current study were to determine priority areas for future research into UK inequalities, and to ascertain suitable methodologies for addressing these inequalities. Key areas of inequality were identified as ‘health’, particularly racial inequalities in health, ‘social care’, ‘living standards’ and ‘economic factors’. A key contribution of the current study was that it addressed the need to ‘assemble evidence on the mechanisms by which policies may affect health’ and will help to ‘provide a framework for the development of new research’ ([73]:290). Uniquely in the current study, participants saw areas of inequality as interconnected and needing to be addressed together. They pointed out that key areas could only be addressed through tackling societal and structural inequalities, chiefly environmental conditions and housing, and having an active prevention programme particularly for young people. Similarly, the Academy of Medical Sciences has argued for a new research paradigm as ‘biomedical research as currently conducted does not have the capacity to address the increasingly diverse and complex issues that transcend disciplinary, sectoral and geographical boundaries’ and recommends taking a ‘much broader view of the drivers of health and the types of evidence we need to intervene’ ([15]:4).

Among the highest and statistically significant priorities for research established by the current study were ‘access to creative and cultural opportunities’, ‘access to nature/outdoor spaces’, ‘sense of place’ and ‘community’. Geographically, the highest priority regions for research determined by the current study were the North East, North West, Wales, and Yorkshire and the Humber. Specific suburban areas, such as those around Glasgow and Belfast, and, more generally, coastal areas in connection with climate change, were also considered likely to benefit from more research. Authors recommend the use of existing high-quality research to support public policy and practice, to ensure local opportunities for people are met, to achieve integration and social cohesion, and to counter place inequality in terms of access and inclusion [50]. Such approaches will require collaboration between community groups, service providers, local authorities, health commissioners, general practitioners and researchers with appropriate longitudinal approaches into the relationship between arts engagement, health and wellbeing [26], required to generate persuasive data. Preventing and tackling loneliness is an important part of these approaches, and will be taken forward by Age UK in the ‘No one should have no one’ campaign, which recognises the importance of improving support for carers, and the crucial need to sustain public services, such as local buses so that older people are not forced to stay at home and risk isolation ([36]:14).

In terms of effects of natural environments on health and wellbeing, it is advocated that ‘widening access to green spaces has to occur in all communities, across the social gradient’ ([14]:126). To ascertain the effects of greater access, ‘future research should follow subjects prospectively, differentiate between greenness quantity and quality, and identify mediators and effect modifiers of greenness-health associations’ ([41]:131) within population level studies [38]. Research investigating income and race inequalities in access to urban green space appears particularly under-developed, with most research on ethnicity and landscape in the UK focusing on rural contexts [55]. Given that a relationship between access to green space and walking was observed for all socio-economic areas, but reduced mortality was only found in the most deprived areas [63], the hypothesised association could be explained by mediators other than walking, such as psychosocial factors, indicating that future research needs to concentrate on understanding causal rather than correlational mechanisms. With the warning that ‘despite the overarching influence of the natural environment on human health, it is the other constructs in these models which continue to receive the greatest amount of attention and study’ ([10]:1016), ecological models of health need to regard the natural environment as fundamental to other constructs. Additionally, it has been pointed out that ‘relationships between sustainable urban environments, public health and social equity can come into a new perspective when viewed through an ecological public health lens’ ([12]: 533).

In terms of suitable methodologies, the current study found a strong emphasis on the use of interdisciplinary mixed methods, including those taken from ethnographic and ecological approaches, and diversification of research to take the views of individual experience into account. The majority of survey respondents selected ‘community/participatory’, ‘action research’, ‘questionnaires/focus groups’ and ‘ethnographic studies’ as suitable methods to research inequalities. Co-participatory and co-production research was highlighted as important, alongside citizen science projects and increased transparency of research procedures. These findings tie in with a key recommendation from the consultation workshop of understanding the community as an ecosystem. Another suggestion was to identify local authorities with stable or narrowing inequality gaps to discover what they did differently, assuming the existence of such authorities. Taking this suggestion further was the idea that joint strategic needs assessments across deprived areas could be carried out specifically to identify issues of health, transport and place-making. These assessments could be used as a basis for offering funding and empowerment to a limited number of UK communities to help them improve health outcomes through an expert facilitation process to enhance social connectedness. Evidence and case studies from these communities could act as a basis for future interventions. The new ‘health of the public’ research advocated six key developments including: developing transdisciplinary research to foster a holistic understanding of the range of determinants of health, and skills and approaches needed to address them, training pathways and professional development; establishing regional hubs to achieve effective connections between practitioners and researchers, ensuring that health and social care services were based upon best evidence; and developing meaningful engagement with all sectors of society ([15]:4). Corresponding with the current study, areas seen to benefit from more research were ‘health/social care including mental health’, ‘advice services’, and ‘adult education/training’.

Responses to Covid-19 restrictions evidenced the importance of ‘community-led responses that draw upon local knowledge and resources, and build capacity and channels of interconnectedness between government, community organisations and the public… those communities that entered the pandemic with such infrastructure have been best placed to respond’ ([65]:7). Coming out of the pandemic, it will be important to address deprivation persisting within communities that has been highlighted and further widened by the Covid-19 crisis; ‘The aim should not be simply to find a way to restore growth of GDP, but to create better societies, characterised by better health and narrower health inequities’ ([4]:141).

### Limitations of the study

A possible limitation is that the research was directly commissioned from the authors to address priorities in health inequalities and how they might be addressed. As such, the scope, budget and time limitations were pre-determined by the commissioners. Consequently, the study used a relatively rapid consultancy process to achieve its aims. Another limitation was that respondent numbers were moderately low, though a similar survey eliciting expert opinion, used fewer respondents over two stages [76]. A further possible limitation was, that although the study attempted to encompass a full range of viewpoints by disseminating the survey link through arts, cultural and natural health networks, there may have been a bias in that a greater proportion of respondents described themselves as ‘academics’. Similarly, there was a relatively low number of workshop delegates invited via a closed list, however the list was compiled to represent a full range of views in the health inequalities field.

## Conclusions

Research demonstrates a clear need to assess the impact of engagement with cultural, community and natural assets on reducing inequality. Research linking the arts, nature and other forms of community engagement with the creation of better societies and the alleviation of inequalities has been centred on small-group studies of extrinsic interventions over a short period of time or large cohort studies of objective factors. Consequently, the evidence base remains patchy, inadequate for decision-making and lacking robustness. There is an urgent need to understand the efficacy of community interventions and how infrastructure and ecosystems research can be applied to all aspects of society, including health, education, employment, and housing, with a view to reshaping it to reduce inequalities in the future. The research carried out here has drawn attention to the proposal that addressing inequalities calls for a new research paradigm that seeks to understand communities from within, by involving local people in action research and by mobilising creative co-productive approaches based upon an ecosystem model. Collaborations between community groups, service providers, local authorities, health commissioners, general practitioners and researchers using longitudinal methods are needed within a multi-disciplinary approach to address societal and structural health inequalities.

## Data Availability

The datasets used and analysed during the current study are available from the corresponding author on reasonable request.

## Abbreviations

APPGAHW: All-Party Parliamentary Group on Arts Health and Wellbeing
CABE: Commission for Architecture and the Built Environment
GDP: Gross domestic product
GP: General Practitioner
ONS: Office for National Statistics
UK: United Kingdom
WHO: World Health Organisation
